# Primary care in the prevention, treatment and control of cardiovascular disease in sub-Saharan Africa

**DOI:** 10.5830/CVJA-2016-082

**Published:** 2017

**Authors:** Dike B Ojji, Dike B Ojji Ojji, Kim Lamont, Karen Sliwa, Olubunmi I Ojji, Bibiana Nonye Egenti, Karen Sliwa

**Affiliations:** Department of Medicine, Faculty of Clinical Sciences, University of Abuja, and Cardiology Unit, Department of Medicine, University of Abuja Teaching Hospital, Gwagwalada, Abuja, Nigeria; Soweto Cardiovascular Research Unit, University of Witwatersrand, Johannesburg, South Africa; Soweto Cardiovascular Research Unit, University of Witwatersrand, Johannesburg, South Africa; Soweto Cardiovascular Research Unit, University of Witwatersrand, Johannesburg, South Africa; Department of Community Medicine, University of Abuja Teaching Hospital, Gwagwalada, Abuja, Nigeria; Department of Community Medicine, Faculty of Health Sciences, University of Abuja, and Department of Community Medicine, University of Abuja Teaching Hospital, Gwagwalada, Abuja, Nigeria; Hatter Institute for Cardiovascular Research in Africa, Department of Medicine, Faculty of Health Sciences, University of Cape Town, South Africa

**Keywords:** cardiovascular disease, treatment, prevention, primary care

## Abstract

Cardiovascular disease (CVD) is the frontrunner in the disease spectrum of sub-Saharan Africa, with stroke and ischaemic heart disease ranked seventh and 14th as leading causes of death, respectively, on this sub-continent. Unfortunately, this region is also grappling with many communicable, maternal, neonatal and nutritional disorders. Limited resources and the high cost of CVD treatment necessitate that primary prevention should have a high priority for CVD control in sub- Saharan Africa. One major challenge of such an approach is how to equip primary care to respond promptly and effectively to this burden. We present a practical approach on how primary care in sub-Saharan Africa could effectively address the prevention, treatment and control of CVD on the subcontinent. For effective prevention, control and treatment of CVD in sub-Saharan Africa, there should be strategic plans to equip primary care clinics with well-trained allied healthcare workers who are supervised by physicians.

## Introduction

The global burden of disease has continued to shift away from communicable diseases to non-communicable diseases, with cardiovascular disease (CVD) taking the lead. The latest Global Burden of Diseases, Injuries and Risk Factors (GBD 2010) study report^1^ shows ischaemic heart disease (IHD) is the leading cause of disability-adjusted life years (DALYs) worldwide, moving from fourth position in 1990 and increasing in incidence by 29%. The same report also ranks stroke as the third leading cause of global DALYs, moving from the fifth position in 1990 and increasing by 19%.

In sub-Saharan Africa, stroke and IHD ranked seventh and 14th, respectively, in the southern region, while they ranked 16th and 20th, respectively, in the western region in 1990. Further projections by the World Health Organisation (WHO)^2^ indicate that by 2030, cerebrovascular and ischaemic heart disease will overtake HIV/AIDS as leading causes of death in this sub-region. It is further elaborated by WHO statistics that by 2030, both cerebrovascular and ischaemic heart disease will contribute to over 20% of the total deaths and 7% of DALYs in sub-Saharan Africa. In South Africa, cerebrovascular disease is the second commonest cause of death after HIV/AIDS, accounting for about 40% of adult deaths.^3^ Unfortunately this sub-continent is still grappling with many communicable, maternal, neonatal and nutritional disorders, which remain dominant causes of the burden of disease.^1^

The health system is therefore overstretched by the existing burden of communicable diseases and a large burden of fastemerging non-communicable diseases, especially CVD. Limited resources and the high cost of CVD treatment suggest that primary prevention should be a priority for CVD control in sub-Saharan Africa.^4^,^5^ One of the major challenges of such an approach is how to equip primary care to respond promptly and effectively to this burden. In this article, we present a practical approach on how primary care in sub-Saharan Africa could effectively address the prevention, treatment and control of CVD on this sub-continent.

## Primary care approach to the treatment and prevention of diabetes and CVD

In spite of the increasing cardiovascular risk burden in sub-Saharan Africa, many healthcare systems in many parts of this sub-continent are designed to treat mostly acute communicable diseases, while neglecting the treatment and prevention of non-communicable diseases, especially CVD.^6^ This is partially due to lack of resources, as the healthcare system in this part of the world is often challenged by lack of sufficient resources and up-todate practical information for healthcare providers.^7^-^9^

Another major challenge to treating and controlling CVD is the large rural population, as there are few providers to serve that population, and distances to the facilities are large, thereby increasing transportation costs.^10^ This is of great concern since the majority of the populace in sub-Saharan Africa resides in rural settings.

Therefore, for there to be any meaningful effect in the treatment and control of diabetes and CVD in sub-Saharan Africa, healthcare must be brought closer to the people. The best way to achieve this is to employ a primary care approach, which should not only be involved in prevention and control but also in treatment of patients. For this type of approach to succeed in sub-Saharan Africa where there is a dearth of physicians, it has to be built around a non-physician workforce, including nurses, community health workers and other allied health professionals in the primary care setting.^11^

In the next section of this review we will discuss the role of the non-physician workforce in this primary care approach. We will also address how the primary care setting can be used to recognise common cardiac problems, such as chest pain, breathlessness on exertion, oedema, palpitations and syncope. The importance of a thorough physical examination as well as relatively simple non-invasive investigations, such as a blood sugar test with a glucometer will be discussed. Furthermore, we will highlight how basic approaches to cardiovascular risk assessment, as developed by the WHO, health professional societies and other expert bodies can be used in the primary care setting. A section of the review will also tackle the important issue of age-appropriate screening for major risk factors, such as hypertension, diabetes mellitus and dyslipidaemia, and address the issue of speciality care referral and long-term primary care management of patients with established CVD and diabetes, using the Seychelles success story.

## Non-physician workforce in primary care management of CVD

Non-physician health workers can be trained in the simple skills of taking a detailed history from patients and good physical examination with the aid of WHO pocket guidelines for the assessment and management of cardiovascular risk.^11^ They should be trained on how to use the WHO pocket guide to recognise people with risk factors who have not yet developed clinically manifest cerebrovascular disease. The effectiveness of this type of model has been demonstrated in Rwanda, where non-communicable disease clinics are run at district level, with such clinics staffed by two or three nurses who see 10 to 20 patients each day.^12^

For effective utilisation of non-physician healthcare workers, they should be trained in the accurate use of basic instruments such as stethoscopes, blood pressure devices, measuring tapes and weighing scales. They should also be able to carry out basic non-invasive investigations such as testing for glucose using a glucometer, testing for albumin in urine using albustix, and blood cholesterol testing using strips. In Rwanda and some other sub-Saharan African countries,^12^ such training is provided at the district level where district hospital leaders, working closely with the staff of healthcare centres, provide in-service training, clinical mentorship and evaluation. Nurses are also trained at district clinics through daily direct patient management while working with physicians.

Another model adopted in this training, which has also been used in Rwanda, involves the use of programme leaders in non-communicable diseases (NCDs), neuropsychiatry and infectious diseases (HIV and TB), who form a chronic care team that trains and mentors a group of healthcare centre clinicians in the basic management of non-infectious diseases.^12^ The main advantage of the use of the programme leader approach is cost effectiveness. In settings such as Rwanda, where there has been success in these training models, training has been formalised into a three-month curriculum in advanced chronic disease management. The effectiveness of such training has been demonstrated in Rwanda where nurses have been trained at the community and district level, not only in basic clinical and laboratory skills, but also in echocardiography for the evaluation of heart failure patients.^12^

The non-physician healthcare worker should also be trained in how to initiate medications such as thiazide diuretics, betablockers, angiotensin converting enzymes inhibitors, calcium channel blockers, aspirin, metformin, statins and insulin. For example, in a model in Rwanda, heart failure treatment initially takes place in district level NCD clinics with non-physicians using algorithms to make the diagnosis and initiate treatments such as frusemide, spironolactone and angiotensin converting enzyme inhibitors.^13^ However, in the use of these medications, the place of regular supervision by a trained physician cannot be over-emphasised. For example, in non-physician-based care of heart failure patients in Rwanda, the role of the cardiologist is restricted to supervision and mentoring of district level clinicians, and evaluation of patients who are potential surgery candidates.^14^

Non-physician clinicians should also be trained to identify serious clinical features in a patient, such as pedal oedema, severe chest pain, and breathlessness on mild exertion and at rest, which are pointers to patients needing referral to secondary and tertiary centres. For example, they should be taught to refer if there are clinical features suggestive of the following: acute cardiovascular events such as heart attack, angina, heart failure, arrhythmias, stroke and transient ischaemic attack, secondary or malignant hypertension, newly diagnosed or uncontrolled diabetes mellitus, and established CVD such as stroke and heart failure.

The non-physician can play a large role in the prevention of CVD and diabetes by using the WHO/ISH risk-prediction charts.^11^ These charts are designed for 14 WHO epidemiological sub-regions and indicate 10-year risk of fatal or non-fatal major cardiovascular events, such as stroke and myocardial infarction, according to age, gender, blood pressure, smoking status, total blood cholesterol level and presence or absence of diabetes mellitus. There are two sets of charts: one set of 14 charts can be used in settings where cholesterol can be measured, while the second set of 14 is for settings where blood cholesterol cannot be measured. Each chart can be used only in countries in the specific WHO epidemiological sub-region.

Before the non-physician health worker applies the WHO riskstratification chart, he/she must select the appropriate chart for the region. The following information should then be recorded: the presence/absence of diabetes mellitus, gender and age of the patient, history of smoking habits, systolic blood pressure and total cholesterol level. These charts are very useful tools that can help non-physician health workers to identify patients with high cardiovascular risk and motivate them, particularly on lifestyle changes and when appropriate, to take anti-hypertensive or lipidlowering drugs and aspirin.

When using these WHO charts, the non-physician health worker must realise that risk stratification is not necessary for making treatment decisions in individuals who belong to the high-risk category, including patients with established CVD, those without established CVD who have a total cholesterol ≥ 8 mmol/l, those with low-density lipoprotein (LDL) cholesterol ≥ 6 mmol/l, those with total cholesterol/high-density lipoprotein (HDL) cholesterol ratios > 8, individuals without established CVD who have persistently raised blood pressure > 160–170/100– 105 mmHg, those with type 1 or 2 diabetes mellitus with overt nephropathy or other significant renal disease, and patients with renal failure and renal impairment.

## Cardiovascular risk prevention at the primary care level

In sub-Saharan Africa, the levels of some risk factors are still relatively low compared to levels in developed nations. For example, many people in rural settings have low-fat diets, regular physical activity and do not smoke.^14^However, the prevalence of other risk factors such as hypertension and diabetes mellitus is of concern; for example, up to 35% of adults aged 25 to 64 years have hypertension.^14^

To prevent an explosion of the growing risks of CVD in sub-Saharan Africa, there must be interventions at the community level, targeting people who do not have established CVD, by reducing risk factors such as high blood pressure, diabetes mellitus and smoking.^15^ To prevent an unhealthy diet, the consumption of local fruit and vegetables should be promoted, as well as reduction of intake of salt, refined sugars and animal fat. People should be encouraged to use vegetables and cereals commonly found in their environment. The promotion of moderate physical activity should be encouraging and inactivity discouraged. Control of diet and physical activity will result in reduction in incidences of obesity, hypertension, high cholesterol levels and diabetes mellitus.

At the primary care level, hypertension is also preventable through a proper diet and physical exercise. A well-tailored hypertension control programme could detect undiagnosed and unregulated hypertensive individuals and thereby significantly reduce the incidence of stroke, heart failure, renal failure and peripheral vascular disease. This could be achieved through regular awareness campaigns in the media, especially on radio and television, and through organised lectures at primary healthcare centres, out-patient departments of secondary and tertiary health care centres, and in community halls, churches and mosques.

## Primary care prevention of CVD: the Seychelles example

A national programme on the prevention of CVD was initiated in 1991^16^ in the Republic of Seychelles, which consists of 115 islands in the Indian Ocean and had a population of 78 846 in 1998. This programme was initiated following an epidemiological survey between 1985 and 1987,^17^ which showed very high rates of cerebrovascular disease (higher than in most European countries) and medium rates of ischaemic heart disease (similar to those in southern European countries) were prevalent in the country, especially in young and middle-aged men. There was also a high prevalence of the classic modifiable risk factors such as hypertension and diabetes mellitus in the adult population, and a substantial proportion of the children were overweight.

The epidemiological survey further attributed the high burden of CVD in Seychelles to dramatic lifestyle changes such as larger consumption of saturated fatty foods, increased intake of salt and calories, and increased prevalence of smoking and sedentary lifestyles.^18^ The dramatic changes in lifestyle were attributed to accelerated socio-economic development and improved standards of living, with the gross national product (GNP) per capita multiplying by 10 (from 600 US$ to 6 000 US$) within 20 years.

Although this programme was started by the Seychelles Ministry of Health-based Unit for the Prevention and Control of Cardiovascular Disease (UPCCD), it progressively involved other sectors such as communities, local parastatals, private companies, international agencies such as the WHO, and academia. The programme was community based, involving non-physician healthcare workers and it was aimed at the promotion of healthy lifestyles and the control of risk factors in the population, in an attempt to prevent and control premature morbidity from CVD, diabetes mellitus and cigarette smoking.

The main approaches used in the Seychelles primary CVD programme included:

Campaigns to raise awareness in the country through the use of media, especially radio and television.
Screening of risk factors in schools through the systematic assessment of body mass index, blood pressure, smoking and other lifestyle habits within routine school medical visits administered to all school children aged five, nine, 12 and 15 years. This screening helped for detection and counselling of children with abnormal reading
Through the World No Tobacco Day programme in the Seychelles, high-profile activities were organised involving large segments of the population, as a main tool for health education on tobacco control. Such events were organised to last several weeks or months, thereby exposing the public to prolonged health education.

Although the Seychelles CVD prevention programme initially relied mostly on health-promotion activities, additional emphasis was progressively put on interventions targeting highrisk individuals, and such measures included the following:

Risk-factor screening in public places and work sites were organised, with a good response. Several companies even provided financial support, so that plasma cholesterol could be measured for all their workers, using point-of-care measurements. In order to ensure diagnosis of high blood pressure and to limit the number of referrals to health centres, screening procedures were extended to include follow-up visits along defined protocols for suspected cases of hypertension, diabetes mellitus and dyslipidaemia.
Health clubs were organised at the community level for high-risk individuals, to encourage them to adopt healthy lifestyles. Sessions were held at primary healthcare centres or in district community centres and were facilitated by staff from UPCCD, the Nutrition Unit of the Health Ministry and local healthcare centres. The sessions focused mainly on skills needed for the adoption of a healthy lifestyle, including demonstrations of healthy cooking. The need for adherence to prescribed pharmacological agents such as anti-hypertensive and oral hypoglycaemic agents was also emphasised.
A register for hypertension and diabetes mellitus was established in 1997. Doctors of all health centres were requested to update a summary form once a year, which was placed in the inner page of the medical file of all patients with hypertension and diabetes mellitus. The form prompted doctors to record one value per year for all major risk factors, including blood pressure, body mass index, total cholesterol, HDL cholesterol, glucose levels, smoking, previous stroke and myocardial infarction. Clerks who filled in the patients’ medical notes then updated a health centre-based register with the information recorded in the forms. Every year, all health centre registers are electronically compiled into a national register in the Ministry of Health. Summary statistics and selected analysis are fed back to health centres and other relevant offices for information and action. For example, the lists of diabetic patients are sent to ophthalmology departments to promote screening and treatment of eye diseases.

Evaluation of the Seychelles primary health prevention programme showed that (1) more than 90% of adults aged 35–65 years were aware of most of the main activities in the prevention programme and a similar high proportion showed good knowledge of CVD; (2) the prevalence of smoking decreased significantly and this was linked to health education and tax increases as a result of strong campaigns against tobacco usage; (3) the active participation of a proportion of the community and the active involvement of key persons and sectors generated a broad coalition among the public, authorities and other organisations that will be very useful in the development of further healthcare interventions and relevant policies; (4) although blood pressure and cholesterol levels increased in the population, it is argued that the levels could have been worse without this intervention programme, bearing in mind the concomitant accelerated socio-economic development.

## Lessons for other sub-Saharan African countries from the Seychelles experience

There are many lessons for countries in sub-Saharan Africa to learn from the Seychelles experience.

The programme was initiated by the government of Seychelles through the health ministry. Other governments in the subcontinent need to play more active roles in the control and treatment of CVD, while not forgetting about communicable diseases. Governments should realise that they can only get support from other sectors such as private, parastatals, academia and international organisations if they are first seen to be committed.
The Seychelles programme was community based, with healthcare brought to the population at their door steps. For any CVD intervention programme to succeed in the subcontinent, a community-orientated approach must be taken, especially with rural areas where transportation is difficult and the population struggles to seek medical help in urban and semi-urban health facilities. Primary healthcare centres need to be equipped with trained non-physician personnel and basic tools such as blood pressure apparatus, glucometers, urinalysis strips and point-of-care machines for cholesterol checks.
The programme succeeded in the Seychelles because it was non-physician based, involving nurses and community health workers, with physicians playing a supervisory role. For a CVD prevention and treatment programme to be effective in sub-Saharan Africa, nurses and healthcare workers, who can reach people at their homes, must be properly trained and empowered. This is also important in regions where there is a lack of trained physicians.
The success of the Seychelles programme can be attributed to good public education. Governments in sub-Saharan Africa need to channel media activities to campaign on the prevention of CVD if we want to curb the coming epidemic. This should be easy in the sub-continent, since much of the media is government owned.
The Seychelles has a comprehensive health insurance scheme for the majority of the population, which ensured success of the preventative measures. This type of programme would be difficult in countries where patients pay out of pocket for healthcare. For any such programme to succeed elsewhere, governments would need to implement comprehensive health insurance schemes.
There was a good referral system in the Seychelles. Governments would need to develop such a system, flowing from primary to secondary healthcare centres and then to tertiary centres.
The Seychelles government was stimulated to embark on the intervention programme as a result of data generated from an epidemiological survey. For countries in sub-Saharan Africa to make any headway regarding prevention of noncommunicable diseases, a non-communicable disease survey is periodically needed in each country.

## Suggested model for the prevention and treatment of CVD and diabetes in sub-Saharan Africa

In view of the success stories in countries such as the Seychelles and Rwanda, the following model may be useful for the sub-continent, with a little modification to suit each country. Firstly, it should be aimed at both the prevention of CVD and diabetes mellitus, and at detecting and treating high-risk patients. Secondly, it should be coordinated by the non-communicable disease units of the ministries of health. Thirdly, it should be community based and involve non-physician health workers such as nurses, community health officers and allied staff, and be coordinated by a physician. Fourthly, there should be an emphasis on primary care in the rural areas.

Primary healthcare centres or clinics should be located near where people live in the rural areas, with a larger comprehensive health centre in the district headquarters, where physicians can supervise the primary health centres. Such primary health centres should be equipped with basic instruments such as weight and height scales, blood pressure apparatus, glucometers, urinalysis strips and strips for cholesterol measurement.

The comprehensive health centres should have an electrocardiography machine. They should be attached to a general hospital to which referrals can be made, while each general hospital or secondary centre should be attached to a tertiary centre or teaching hospital. Furthermore, there should be annual reports for each primary and comprehensive health centre, and performance should be rewarded.

Finally, governments should take the lead and involve the private sector, non-governmental organisations and academia. Such projects require constant funding and support.

## Conclusion

The high burden of CVD and diabetes mellitus in sub-Saharan Africa means that individual governments must develop strategic plans to equip their primary care clinics with well-trained non-physician healthcare workers and basic instruments, such as blood pressure apparatus, kits for urinalysis, and blood glucose and cholesterol checks if an epidemic of chronic non-communicable diseases is to be averted on the sub-continent. Governments should also set up a referral system whereby there is supervision and referral from primary care to secondary centres, and two to five primary care centres should be attached to a secondary centre.

**Fig. 1.**
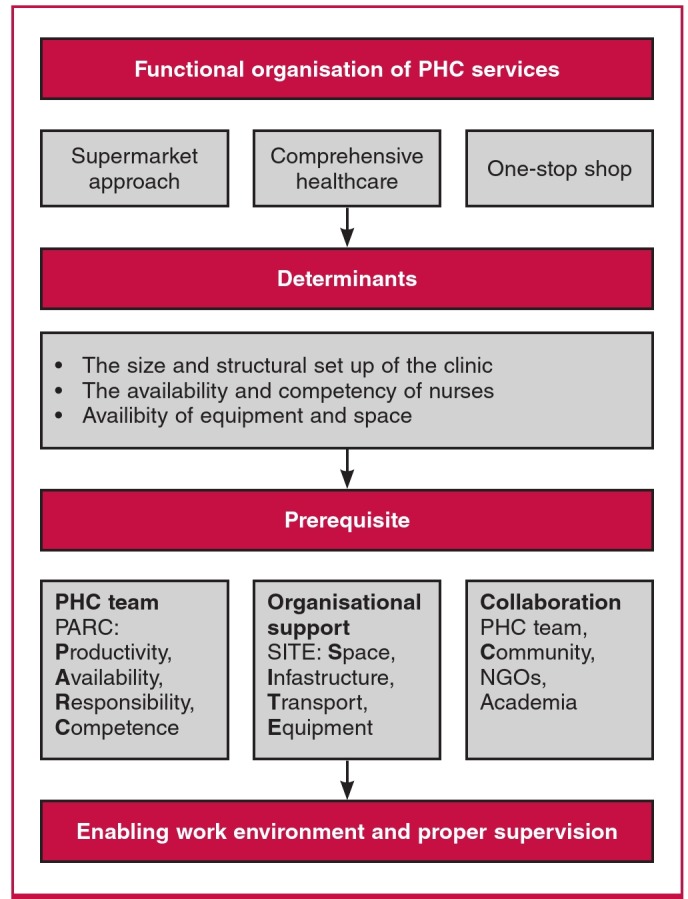
Organisational structure showing how primary healthcare can be effective in preventing cardiovascular disease. PHC = primary healthcare. NGOs = nongovernmental organisations.

The Seychelles experience shows that the key drivers of effective primary care to yield population-level impacts on diabetes mellitus and CVD include research, a non-physicianbased approach, and integration of the media, the general population, academia and non-governmental organisations. For such a programme to succeed there must be a holistic framework, developed by the government and coordinated by the NCD unit of the federal ministry of health, as shown in Fig. 1. It should involve research on CVD and diabetes, structured training of non-physician healthcare workers, appropriate equipping of primary healthcare centres and correct referral systems from primary to secondary centres.

Limited resources were a disadvantage in the Seychelles programme and would also be in sub-Saharan Africa. This could be overcome with the support of non-governmental agencies and the private sector, however, they would invest in such programmes only if they had confidence in the government of the country.

**Key points:** 

CVD plays a leading role in the disease spectrum of sub- Saharan Africa, with stroke and ischaemic heart disease ranked as seventh and 14th leading causes of death, respectively, on this sub-continent.
Limited resources and the high cost of CVD treatment necessitate that primary prevention should have a high priority for CVD control in sub-Saharan Africa.
For any CVD intervention programme to succeed on the sub-continent, a community-orientated approach must be taken, especially in rural areas where transportation is difficult, deterring people from seeking medical help at urban and semi-urban health facilities.
Primary health centres therefore need to be equipped with trained non-physician personnel who are supervised by physicians, and also with basic tools such as blood pressure apparatus, glucometers, urinalysis strips and point-of-care machines for cholesterol checks.

## References

[1] Murray CJL, (2012). Global Burden of Disease study group 2010. Disabilityadjusted life years (DALYS) for 291 diseases and injuries in 21 regions, 1990–2010: a systematic analysis for the Global Burden of Disease study 2010.. Lancet.

[2] Mathers CD, Loncar D (2006). Projections of global mortality and burden of disease from 2002 to 2030.. PLoS Med.

[3] Opie LH, Mayosi BM (2005). Cardiovascular disease in sub-Saharan Afric. Circulation.

[4] (1982). WHO Technical Report Series No 678.. World Health Organization, Geneva.

[5] Chockalingam A, Balaguer-Vintro I, Achutti A, de Luna AB,, Chalmers J, Farinaro E (2000). The World Heart Federation’s white book: Impending global pandemic of cardiovascular diseases: challenges and opportunities for the prevention and control of cardiovascular diseases in developing countries and economies in transition.. Can J Cardiol.

[6] Yach D, Hawkes C, Gould C, Hofman K (2004). The global burden of chronic diseases: overcoming impediments to prevention and control.. J Am Med Assoc.

[7] Addo J, Smeeth L, Leon DA (2007). Hypertension in sub-Saharan Africa: A systematic review.. Hypertension.

[8] Motala AA (2002). Diabetes trends in Africa.. Diabetes Metab Res Rev.

[9] Whiting DR, Hayes L, Unwin NC (2003). Diabetes in Africa. Challenges to healthcare for diabetes in Africa.. J Cardiovsc Risk.

[10] Goudge J, Gilson L, Russel IS, Gumede T, Mills A (2009). Affordability, availability and acceptability barriers to healthcare for the chronically ill: longitudinal case studies from South Africa.. BMMC Health Serv Res.

[11] (2007). Pocket Guidelines for the Assessment and Management of Cardiovascular Risk..

[12] Bukhman G, Kidder A The partners. In: Health Guide to Chronic Care Integration for Endemic Non-Communicable Diseases..

[13] Kwan GF, Bukhman AK, Miller AC, Ngoga G, Mucumbitsi J, Bavuma C (2013). A simplified echocardiographic strategy for heart failure diagnosis and management within an integrated non-communicable disease clinic at district hospital level for sub-Saharan Africa.. J Am Coll Cardiol.

[14] (2005). Cardiovascular diseases in the African region: current situation and perspectives.. Report of the WHO Regional Director.

[15] Stewart S, Sliwa K (2009). Preventing CVD in resource-poor areas: perspectives from the ‘real-world’.. Nat Rev Cardiol.

[16] Gervasoni JP, Bovet P, Shamlaye C, Paccaud F (1991). Guidelines for a collaborative long-term programme of reduction of cardiovascular risk factors in the population of the Seychelles.. SozPraventivmed.

[17] Bovet P, Perret F, Shamlaye C, Darioli R, Paccaud F (1997). Seychelles Heart Study II: methods and selected basic findings. Seychelles Med Dent J.

[18] Bovet P, Shamlaye C, Kitua A, Riesen WF,, Paccaud F, Darioli R (1991). High prevalence of cardiovascular risk factors in Seychelles (Indian Ocean).. Arterioscler Thromb.

